# Modelling social networks for children of parents with severe and enduring mental illness: an evidence based modification to the network episode model

**DOI:** 10.1186/s40359-024-01647-3

**Published:** 2024-03-19

**Authors:** Imogen Nevard, Helen Brooks, Judith Gellatly, Penny Bee

**Affiliations:** Division of Nursing, Midwifery & Social Work, Faculty of Biology, Medicine and Health, School of Health Sciences, The University of Manchester, 6.333 Jean McFarlane Building, Oxford Road, Manchester, M13 9PL UK

**Keywords:** Vulnerable children, Mental ill parents, Social networks, Social network analysis

## Abstract

The Network Episode Model (NEM) is a well utilised model conceptualising how social networks, valuable resources which can positively impact wellbeing and functional outcomes, are responsive to the needs of people with physical and mental health difficulties. Children of parents with severe and enduring mental illness (COPMI) are impacted by these illnesses through the intersecting roles of kin relation, informal carer, and dependent. However, it is not clear that social networks effectively respond in kind to the child’s episodic need.

We draw upon qualitative data to propose a new multi-factorial conceptual model (COPMI-NEM), triangulating parental mental illness, child’s developmental stage and social ties to theorise how social networks do respond and adapt to children’s needs.The model illustrates how networks are typically flexible and responsive to visible age-related needs, but less reactive to more obscured needs. Successful network navigation and negotiation of support relies on multiple factors including the child’s ability to successfully activate social ties and the availability of formal networks. We propose a new theoretically and empirically informed NEM model also available for testing, refinement and validation in other young carer populations exposed to episodic parental health needs. Findings from this study could be used to generate network informed interventions for this and comparable populations.

**Supplementary Information:**

The online version contains supplementary material available at 10.1186/s40359-024-01647-3.

## Background

The Network Episodic Model (NEM) of health has been widely adopted in the conceptualisation of chronic, episodic health conditions, including severe and enduring mental illness (SMI), for many adult and some child populations [1–6]. Currently this analysis has not been extended to consider dependents of adults with episodic health related needs. Network theory for vulnerable adult populations highlights the dynamism of network relations and complexity of navigation for vulnerable adults, evidenced through qualitative research examining individual-level networks [7]. Similar research is required in child populations, to adequately account for complex network structures and negotiation dynamics, incorporating additional factors including limited resource access, autonomy and cognitive capacity compared to adult populations. This paper presents an adapted theoretical network model for children of parents with severe and enduring mental illness (COPMI) grounded in qualitative research with under 18s.

COPMI are a vulnerable child group at risk of a number of adverse outcomes, including psychiatric disorder, poor physical health and increased healthcare usage, welfare concerns and negative behavioural, educational and psychosocial outcomes, including social isolation [8–15]. International systematic reviews find that over half of adults with SMI are parents, as are approximately a third of adults accessing inpatient or community mental health care [16]. Recent systematic reviews show that parents with SMI can experience a constrained parenting style and parenting difficulties; inpatient admission particularly impacts parenting and connections for support are desired [17, 18]. The COPMI-NEM model presented below provides a theoretical account of the operation of these network connections.

A social or personal support network is a personal community: the set of active and significant ties which are important to an individual’s everyday life [19]. Social ties serve a number of health and wellbeing related functions, including access to instrumental, emotional and informational support. A recent systematic review on the social networks of vulnerable children has shown that vulnerable children and young people commonly have impoverished networks. Network embeddedness (i.e. the availability and utilisation of this set of active, valued and functional ties) is associated with positive health, wellbeing and functioning outcomes. Family and parents are typically primary providers of support, but ties can be substituted when networks are restricted. There is in general a dearth of explanatory data demonstrating the causal mechanisms between network embeddedness and positive outcomes for vulnerable children. A lack of theoretical modelling is also apparent, critical to conceptualising the role of vulnerability characteristics in shaping personal networks [20].

Network research for COPMI populations is limited [21]. Informal networks are the primary vehicle by which children meet their physical and psychosocial needs (through kin ties and peer relationships). Social network models of typical child to adolescence development indicate that children begin to transition from dependence on adults to independence between the ages of 10 and 13 [22]. This independence is characterised by increased reliance on peer groups in place of family members. A COPMI network model that locates the central role of networks to child wellbeing will need to account for both dependant developmental stages and increasingly independent stages. At all stages, the ill parent may episodically experience limited parenting capacity; networks are therefore an integral vehicle of social support which facilitate access to emotional, instrumental and information support. COPMI are under identified by statutory services so likely to be more reliant on informal networks for support [23–25].

The NEM conceptualises help-seeking as a process mediated by social networks. Four domains inter-relate to determine an individual’s access to support in response to their needs: (i) illness career (fluctuating characteristics of health engagement), (ii) social support system (informal networks) and (iii) treatment system (formal networks) and (iv) social context (personal, health, and psychological characteristics of the individual). The model is grounded upon four fundamental premises regarding the nature of social actors in terms of resource and decision making. Children occupy a different social and developmental position to adults, so these assumptions are recontextualised as an axiomatic basis for applying this model to this population, detailed in Table 1 [5]. The adapted COPMI-NEM provides an empirically informed framework for conceptualising COPMI networks, necessary for illuminating the hidden needs of this population and allowing for theoretically informed future interventions, a priority for social relationship interventions in mental health fields [26].

**Table 1:** Assumptions underpinning the NEM and adaptions required for COMPI-NEM [5]

Assumptions	COPMI Factors
Societies hold a vast reserve of people who can be and are consulted during an illness episode i.e. a multiplicity of resources with the potential for therapeutic function	The visibility of a parental episode in COPMI networks is likely to vary; there is an additional degree of separation between the parent exhibiting mental health need and network to be activated via the child [23, 27] (Ofsted, 2013; Gray et al., 2008)
Bounded rationality (decision makers are rational, but limited by contextual factors such as time, information and cognitive capacity)	Children face unique contextual factors and bounds to rationality compared to adults, such as reduced relative cognitive capacity and reduced autonomy in type and variety of social environments exposed to
Decision making is dynamic; decision makers form strategies, which are informed by consequences of previous decisions	Children have limited prior data to strategy form; children are actively learning decision making autonomy [28]
Decision making is informed by interaction with social ties which form networks	This may be particularly true for children with lower relative decision making autonomy and increased dependence on caregivers [28]

## Methods

### Aim

This study aimed to generate inductive findings regarding the phenomenological experiences of COPMI based on an analysis of child generated (rather than proxy generated) data. This study aims to provide a qualitative description of the characteristics, role and function of social networks for COPMI based on an inductive thematic analysis of themes designed to generate network features.

### Sample

Inclusion criteria comprised: (i) aged 6-17yrs (ii) minimum 10 h of contact per week with (iii) a parent with a SMI, defined as a psychiatric diagnosis of chronic symptomology (minimum one year duration) with significant impact on daily functioning. The sample was recruited via advertisement in third sector organisations working with this population, such as young carers groups. Participants either responded directly to a physical or digital advertisement, or were approached by the organisation due to their eligibility for participation. Table 2 details sample characteristics.

**Table 2:** Participant Information

Gender	(*n*)%
M	(6) 35.3
F	(11) 64.7
Other	0
**Ethnicity**	**(*n*)%**
White British	(16) 94.1
Other	(1) 5.9
**Age**	
Mean (SD)	10.41(3.66)
Range	6–17

An information pack detailing the aims, involvement expectations and protocols of the study was distributed to families in advance of interviews by the recruitment organisation. The interviewer was introduced to children immediately prior to interview, where the role of the researcher, the process of interview and the purpose of data collection was described to families or individuals by staff and the interviewer.

### Data collection

**Fig. 1:**
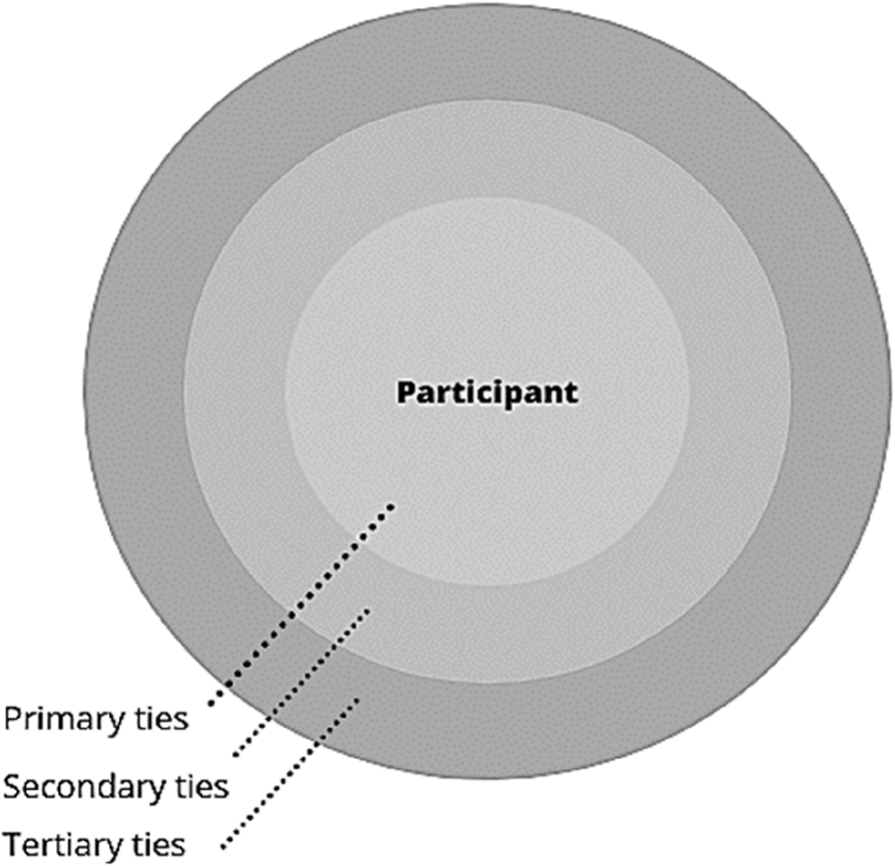
Template Ego-Net Diagram

A single researcher (IN) conducted 17 thirty minute, semi-structured network interviews across three third sector sites in England with children who fit the inclusion criteria. These qualitative interviews created for this study, incorporating personal network mapping, were conducted one on one with children (see online electronic supplemental appendix 1). Under 16s had the option to have a parent present if preferred by the child or parent but none expressed this preference. All interviews were audio recorded. Children were asked to identify a range of social ties, ranked into three tiers of importance; a template ego-net diagram is shown in Fig. 1. Flexibility was permitted regarding who or what constituted a network tie (animals, toys and valued activities were often reported) as recommended in current qualitative methods in social network research [29].

Interviews were completed at the site of the third sector organisation aiding recruitment between August 2018 and March 2020. All interviews were conducted by the first author, who is both a counsellor and researcher. The interview process was piloted in advance of this data collection process and the interviewer had no pre-existing ties to the children or recruiting organisations prior to this project.

The authorial team agreed a consensus that data was approaching saturation at 14 interviews due to consistent repetition of key themes. Research concluded at 17 interviews, due in part to data collection limitations during the Covid-19 pandemic. There was no opportunity for respondent validation, due to public health related closure of recruitment organisations.

### Data analysis

The NEM was adapted and refined using a general inductive approach [30]. Interviews were transcribed verbatim by the interviewer and compiled alongside network matrices using NVivo.12 [31]. This document was distributed amongst the research team for data immersion and familiarisation. Raw data was coded by the interviewer and annotated transcripts were distributed between researchers for agreement. The research team as a whole then reviewed the coded transcripts, and grouped codes into categories. Categories were developed into a framework of key themes and processes by the research team, through group consulted comparison, physical mapping and reconfiguration.

At this point, parallels to the NEM were identified. Each feature of the original model was triangulated against the original data and the conceptual framework created by the preliminary thematic analysis. These features were compiled and mapped against the original model, generating the revised COPMI-NEM model described in results. Consistencies and divergencies between the original and revised model were contrasted by the team, and a rationale for each was considered based on the original and coded data. The final framework was appraised and agreed upon as representative of the data set by the study team.

### Ethical considerations

There are no conflicts of interest between the study team and third sector organisations to report. This study was approved by the University of Manchester Research Ethics Committee (Ref: 2018–3572-6390) and Barnardo’s Research Ethics Committee (approved 16/07/2018). This research project was based in the University of Manchester and part-funded by the NSPCC.

## Results

The data analysis process found that the NEM has conceptual relevance for this population. Participants described dynamic and intermittently responsive networks, a key theoretical premise of the original model. As hypothesised, children of these adults reported similar fluctuations in need related partially to parental illness patterns as well as personal needs, and dynamic shifts in the support provided through network connections.

**Fig. 2:**
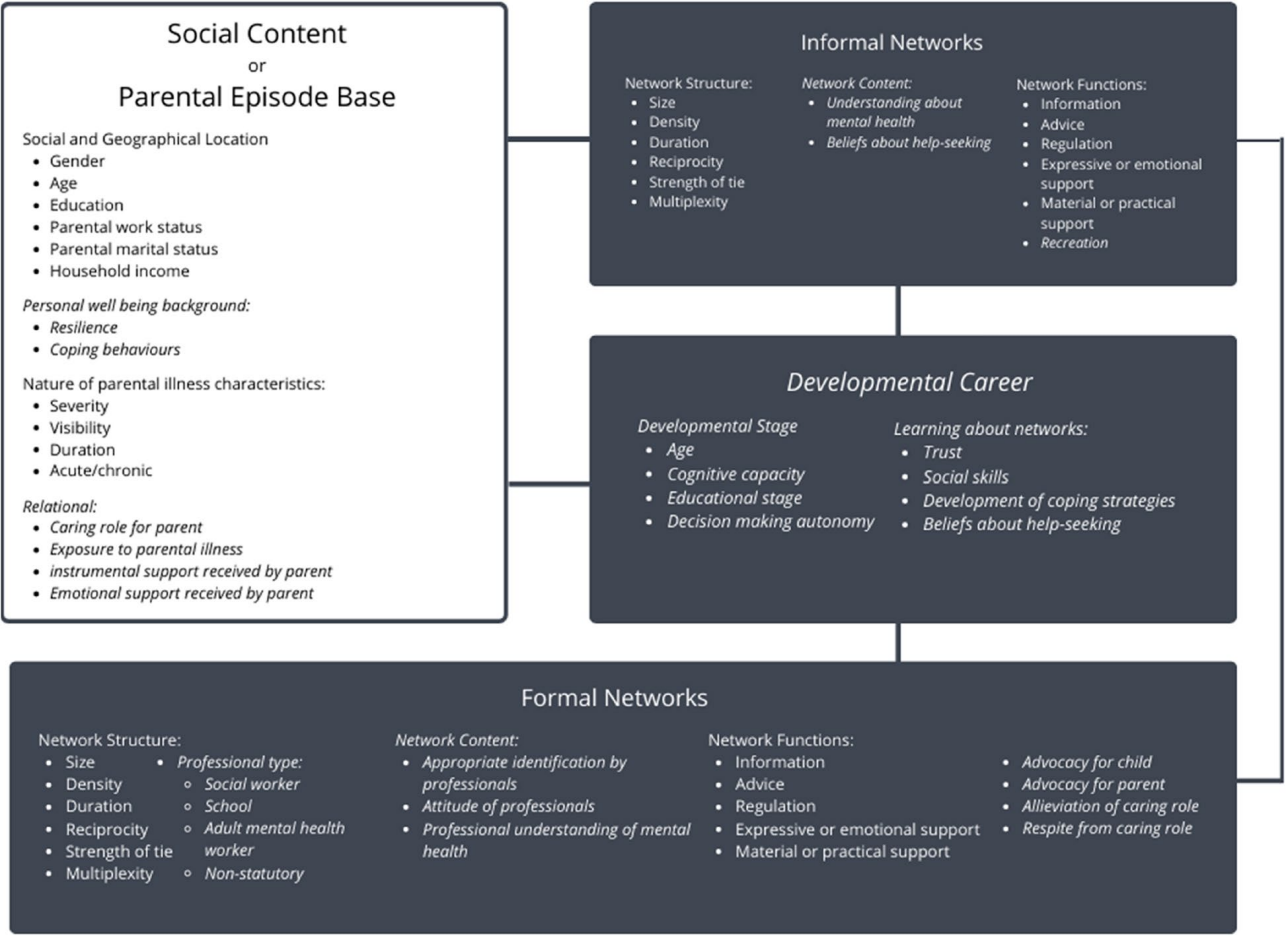
COMPI-NEM (adaptions italicised)

Figure 2 presents a proposed adapted network model describing these network fluctuations, resultant support flows and dynamic shifts over time for COPMI.

### COPMI-NEM adaptations

#### Developmental career substituted for illness career

Substitutions, additions and adaptations have been made to the model in order to reflect the dynamic network changes described by participants. Children reported dynamic shifts in network-derived support over time where networks were more responsive to children’s age related needs than to needs related to parental illness. Illness career was substituted for developmental career as the central longitudinal feature for contingent network activation in this revised model. Young children frequently reported instrumental support from family members and family friends including being looked after overnight or for periods of time, a situation less reported by older participants. Older children described a reduction of this type of support from family and other adults over time.

Severity of parental illness episode impacted the child’s level of strain in a non-linear course. Increased child independence or competence was described in tandem with increased provision of instrumental support to parent by the child, alongside decreased receipt of instrumental support, in a linear trajectory. Children first report this change of providing more and receiving less instrumental support around the start of secondary school, a time often showing marked changes in levels of independence.

Another component of developmental career is learning, including that about network navigation and coping strategies. Current research on adult networks indicates that network activation is an active process requiring significant relational work on the part of the individual [32]. Participants in this study reported varied levels of network activation and different network navigation strategies particularly in peer or informal relationships; previous research indicates that friendships are a priority area for COPMI [25]. Multiple reasons were given for this, including difficulty making friends, changes to schooling, or an active decision to avoid social ties. These learnt coping strategies in response to network strain will have an impact on future network navigation. Learning regarding networks over developmental career is likely to incorporate trust, beliefs about help-seeking and the evolution of varied social skills and coping strategies.

#### Social content and parental episode base

Most social or geographical demographic features described in the social content NEM domain are equally applicable to this adaptation. For example, parental marital status, parental work status and household income will impact the child but be determined by other members of the family unit rather than the child themselves. Similarly, the illness characteristics of the parent will be a relevant influencing factor on the child, and resultantly their networks.

The individual’s health background has been substituted for the child’s wellbeing background including internal characteristics such as resilience and coping behaviours employed. A further component, relational context, has been added to this section. COPMI are a population of interest not because of an internal characteristic but due to a filial relation to an adult with an identifying characteristic of mental illness. This relational context will underpin many of the child’s needs with respect to network resources including the caring role the child has taken on for their unwell parent as well as the instrumental and emotional support the ill parent receives from other sources. The level of exposure the child has to their parent’s illness, including whether they live between multiple households, and how involved they are in their parent’s healthcare and treatment programme all contextualise the child’s level of need.

#### Formal networks

Children will engage with professionals from a range of statutory and non-statutory services including social workers, teachers and educational professionals, their parent’s treatment team, and other organisations aimed at supporting children with additional need, this latter reliant upon appropriate identification, often limited by barriers to family focused practice, concerns about scope and remit, low levels of interdisciplinary communication and minimum thresholds for service provision, as well as limited family communication with the child around parental SMI for perceived protective reasons [33–35]. A child may need to exhibit significant educational, behavioural or psychosocial difficulties for this to apply; even in this context parental mental illness is often poorly assessed [36, 37].

As addition to initial identification, successful network activation subsequently requires compassionate attitudes and adequate understanding on the part of these professionals. Prior NEM formulations for other vulnerable child groups have similarly identified the need for sensitive approaches from first formal identification onward3. The children in this sample, despite being recruited through services that had identified them as having additional support needs, did not always report successful identification from the professionals around them, particularly in school settings, a common finding in research with this population [23].

Formal networks provide similar functional roles for these children as described in the original model such as information, advice, and practical and emotional support. Functions identified by COPMI included advocacy work for the family, and targeted intervention or respite designed to reduce their caring role. Children who had been identified in educational settings as in need of additional assistance or who were in receipt of appropriate advocacy, flexibility and emotional and instrumental support reported positive educational experiences. However, children and young people who had not been identified in this way reported the inflexibility of educational systems to their particular needs as a source of acute strain.

Older children who reported strong and effective formal networks often described professionals as contributing to wellbeing in provision of instrumental and informational support in the same time developmental period as informal networks diminished over time. Organisational links also facilitated access to emotional and recreational ties.

#### Informal networks

The informal network domain requires little adaptation from the original model. Structural factors will be of similar interest, although structures are likely to differ considerably from adults. Network content is likely to inform the child’s understanding of the situation, and thus their ability to activate networks around them, including knowledge levels around mental health, and beliefs about help-seeking. A depth of mental health understanding and positive attitudes to help seeking within the child and their broader networks are likely to increase the efficacy of both informal and formal networks. Participants overall demonstrated limited mental health literacy, or understanding of terminology surrounding their parents illness.

Network function is conceptualised as in the original model, although importance of relative functions may vary; for example, young children will be heavily reliant on instrumental support from others. Similarly, young children may be less able to actively influence the composition of networks around them, reliant on dependent structural factors such as location, family composition and school system. An additional function of networks included in the COPMI-NEM is recreation, play representing a significant aspect of their child social ties. Non-human network members were prioritised as a primary-level tie (principally animals but also toys and valued activities) by almost every child. Pets were reported to play meaningful roles in companionship, play and emotional regulation.

A diversity of functional requirements here indicates that a diversity of network member types are needed to meet COPMI’s varied instrumental, emotional, informational and recreational needs. These include basic survival needs, need to perceive care and love from others, need for information about parental mental health and general life skills and play partners.

## Discussion

The NEM is an appropriate model to describe the inter-relation of social determinants, formal and informal networks and parental illness characteristics in COPMI networks. Modification is required to incorporate both relevant parent and child variables, most notably child developmental stage and associated levels of autonomy and need. Informal support diminished over time with developmental career, hypothesised to relate to expectations of independence by network members. This is problematic for children who may find their support needs increase with time. Caring role does not automatically activate informal network mobilisation, explicable through limited visibility of needs. We can infer that help-seeking behaviours are pivotal.

Social networks are at times a source of strain, especially related to stigma around parental illness and trust, a factor commonly linked to social capital measures in network analyses [38]. Children exposed to negative experiences described either strategic navigation of ties in future, or avoiding peer connections altogether; prior reviews show that stigma related to parental SMI is associated with social isolation [39]. Prior research indicates that when COPMI employ avoidant coping strategies they are more likely to develop later onset mood disorder [40]. This illustrates decision-making informed by prior experiences described in the original model. Network shrinkage can be positive for adults with chronic health conditions [41, 42]. However, if children use this strategy, there is a risk that children are permanently separated from their peers or adults, inhibiting future help-seeking. Vulnerable children exhibit impoverished networks; this theoretical model suggests COPMI networks have the potential to degrade over time, representing a crucial intervention point.

Children are sometimes but not always adequately identified by formal networks. A number of children in this study, despite being recruited through third sector organisations aware of their COPMI status described a lack of formal support more broadly from educational or statutory professionals. Formal and informal networks can provide compensatory ties where children exhibit additional need. Formal networks may be particularly important in cases where there are insufficiencies in informal networks, either due to limitations in network size or lack of network responsiveness to need. Children take both active and passive roles in network processes as their decision making autonomy develops over time. Examples of active behaviours include intentional network navigation or help-seeking behaviours; passive roles include situations where children rely on family networks or formal systems to identify their need.

### Application

The aim of this study was to create an evidence-based theoretical model rather than generate recommendations for application. However, we present here some brief considerations regarding optimal integration of these findings into health and social interventions and clinical practice. Relevant findings to inform application of research include (i) the identification of crucial chronological stages in network change over a child’s developmental career (specifically ages 10–13), (ii) the substitutability of formal network ties for diminished informal social ties, (iii) the use and development of network navigation and activation strategies on the part of the child and (iv) the potential for long term network shrinkage if these strategies are not developed and integrated into the child’s interpersonal developmental learning.

Research shows that adults typically mobilise network resource well and actively, dependent on such factors as culture, availability and individual resource [43]. This is harder for children to do so, so responsibility for identification and proactive support needs to be devolved outward into diverse networks. For this reason, meaningful ties with professionals in network is descriptively associated with positive outcomes, and vice versa. Services and organisations that come into contact with this child group, most notably adult mental health workers, educational professionals, statutory and non-statutory workers should make a concerted effort to identify COPMI before nonadaptive coping strategies that inhibit network activation are cemented. Additional screening at the educational transition between primary and secondary education could present opportunities to identify these often hidden children at a pivotal stage in their psychosocial development.

Post-identification, interventions could incorporate two components, reflecting both the passive and active roles children take in network navigation. Conceiving of children as active subjects challenges a prevalent discourse that construes children as passive, unfinished persons, a criticism levelled at theories conceptually framed only in terms of risk and resilience [44]. First, interdisciplinary collaboration focussed on creating robust formal networks that support child need between health, educational and other professional settings will increase opportunities for access to instrumental, emotional and informational support that may become lacking in informal networks over time. Older children who described supportive formal networks in this study noted the collaboration between school, third sector advocates and mental health professionals as significant in navigating the particular challenges they faced. Young people reporting negative experiences described rigid systems that did not afford them the flexibility to navigate their fluctuating needs.

Secondarily, interventions incorporating the child’s newly developing decision making autonomy over the developmental career are likely to focus on equipping the child with the internal skills that will assist them in help-seeking behaviours and network activation in the future. This style of intervention is likely to be most successful prior to potential network shrinkage, but could add utility throughout a child’s developmental career, as network embeddedness is associated with positive wellbeing up to and into adulthood [20, 45].

Skill development programmes for this population could focus on awareness raising of available formal network ties available to them, improvement of mental health literacy to assist in making informed choices when navigating social stigma of parental illness in these networks. Peer relationships can also represent a source of strain for these children [21] and the role of social stigma related to parental illness as an inhibitor of network growth, activation and navigation has repeatedly appeared throughout this study. Therapeutic support geared toward helping the child beneficially integrate negative experiences into their future coping approaches with particular respect to network navigation could improve child wellbeing. Interventions targeted at improving the child’s autonomous network activation ability could operate at an individual or family level.

### Limitations

This study has a number of potential limitations. A lack of ethnic diversity in the sample represents a difficulty in extrapolating findings across populations. This was identified by the research team at the time of data collection, however the dependence on intermediary recruiters in sampling limited influence over sample demographics. The use of intermediary organisational recruiters also meant that all children were in receipt of some third sector support, so were unlikely to include the most vulnerable subsections of this demographic. However the study purposefully recruited from a range of organisations, not just young carer groups, so represents a more diverse group than some comparable research.

No children under 6 were included in this study due to the methodological limitations of social network interviewing make it challenging to collect data from children at earlier stages of life course. The validity of this model could have been further evidenced through a process of respondent validation, or further interviews with additional children to elicit feedback on preliminary themes identified. However, opportunities for respondent validation were limited due to closure of recruitment organisations during the Covid-19 pandemic.

### Further research

There are a number of avenues for future research building upon the theoretical contribution of this study. Further data collection with representative samples of the target demographic of this study will help ascertain the appropriateness of findings across the population as a whole. Further data collection could also improve specificity regarding time frame and pivotal stages in children’s developmental career. This is a framework that not only has potential to inform social interventions for COPMI, but one that could with some adjustment be extrapolated into social interventions for young carers as a whole. When contextualised in empirical data that sees increases in adult multi-morbidity across all ages, as well as CYP care load mounting particular in response to the recent pandemic, the coming decade could represent a pivotal time for investing in these interventions [46–49].

Consultation with professionals and clinicians in related fields will be constructive in developing appropriate interventions that could be integrated into their work with this demographic. Furthermore, feedback on how they integrate social network perspectives into their practice overall will help inform the future application of theoretical work.

## Conclusions

The COPMI-NEM represents an evidence-based adaptation of the network episode model. This model is useful for clinical practice when generating network-informed interventions for this demographic. Formal networks should aim to identify children early (before 10–13), to provide them with a wealth of professional ties to supplement instrumental, emotional and information support that may be lost with informal network shrinkage over time and to help children develop positive network navigation and activation strategies including help-seeking behaviour, mental health literacy and peer interaction skills.

### Supplementary Information

**Supplementary Material 1.**

## Data Availability

The data that support the findings of this study are not openly available due to reasons of participant confidentiality. Data are located in controlled access data storage at the University of Manchester.

## Abbreviations

COPMI: Children of parents with mental illness
SMI: Severe and enduring mental illness
