# Supplementary material for: Modelling social networks for children of parents with severe and enduring mental illness: an evidence based modification to the network episode model
Source: BMC Psychol. 2024 Mar 19;12:162. doi: 10.1186/s40359-024-01647-3 (PMC10949563; doi:10.1186/s40359-024-01647-3)
Supplement: Supplementary file 1 — Supplementary Material 1. [file 40359_2024_1647_MOESM1_ESM.docx]

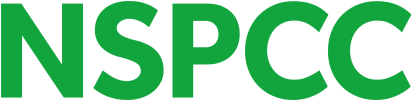

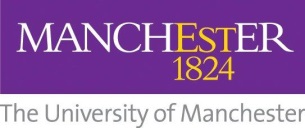


**Interview Topic Guide**

General prompts

**I’d like to know about the people, places, activities and things that are important to you. The most important go in the middle, the almost as important in the second circle, and the third most important go in the outside circle.**

Follow up

**What does this person do? How do they matter to you? Why did you put them in this circle?**

**What does each place/activity involve? What’s important about it? Why did you put it in this circle?**

Name generator prompts if necessary

**Who would you talk to about something really important?**

**If you were feeling sad, who would you want to be with? Who would be your second choice?**

**If you were worried about something, who would you ask for help?**

**If you had some really good news, who would you want to tell? Who else?**

**If you wanted to have a party of all your favourite people, who would you invite first? Who else?**

**If you needed help, who would you go to?**

Dynamic shifts

**Is there anyone/anything who used to be in this map, but isn’t any more? Why not? How it is different now compared to when they were around?**

**Has anyone/anything just recently become important for the map? How is it different now compared to when they weren’t around?**

**Is there anyone who could be in the map, who might not be important in a good way?**

**What would you change? Would you change things?**
